# Machine learning–based ecological prediction of maternal mortality ratio in malaria-endemic Africa

**DOI:** 10.3389/fpubh.2026.1929496

**Published:** 2026-09-28

**Authors:** Messaouda Belfakir, Imad Shahid, Mohamed Belfkir, Balázs Ádám, Rami AlRifai, Farida Alhosani, Abdessamad Tridane

**Affiliations:** 1 Institute of Public Health,College of Medicine and Health Sciences, United Arab Emirates University, Al Ain, United Arab Emirates; 2 Mechanical and Aerospace Engineering Department, College of Engineering, United Arab Emirates University, Al Ain, United Arab Emirates; 3 Physics Department, College of Sciences, United Arab Emirates University, Al Ain, United Arab Emirates; 4 Infectious Diseases Epidemiology Research Advancement Unit (IDERA), Institute of Public Health, College of Medicine and Health Sciences, United Arab Emirates University, Al Ain, United Arab Emirates; 5 The Global Institute for Disease Elimination, Abu Dhabi, United Arab Emirates; 6 Mathematical Department, College of Sciences, United Arab Emirates University, Al Ain, United Arab Emirates

**Keywords:** Africa, governance, institutional capacity, machine learning, malaria, MMR, SVR

## Abstract

**Background:**

Malaria in pregnancy remains a significant public health concern in sub-Saharan Africa. It may contribute to population-level maternal mortality through adverse maternal outcomes. This study aims to develop a machine learning framework to predict maternal mortality ratio (MMR) across African countries.

**Methods:**

A supervised machine learning framework was applied to identified open source, country-level data across 52 African countries through (2000–2020). MMR was estimated using Support Vector Regression (SVR) model. Model performance was evaluated using a random 70/30 split of country-year observations, temporal block sensitivity analysis applied using 2000–2016 for training and 2017–2020 for testing and 5-fold grouped cross-validation in which entire countries were held out from model training.

**Results:**

MMR was positively correlated with stillbirth rate (*r* = 0.76; 95% CI, 0.65–0.84), total fertility rate (*r* = 0.65; 95% CI, 0.51–0.76), malaria incidence (*r* = 0.42; 95% CI, 0.24–0.58), female malaria prevalence (*r* = 0.41; 95% CI, 0.22–0.58), and anemia prevalence in pregnant women (*r* = 0.38; 95% CI, 0.20–0.55). Inverse correlations were observed with HDI (*r* = −0.75; 95% CI, −0.83 to −0.63), female primary completion (*r* = −0.67; 95% CI, −0.79 to −0.52), and female secondary enrolment (*r* = −0.68; 95% CI, −0.77 to −0.57). Temporal-holdout permutation importance identified HDI and stillbirth rate as the leading contributors to predictive performance, whereas female malaria prevalence and malaria incidence had smaller positive importances. Country-specific *R*^2^ values ranged from −0.202 to 0.891, indicating substantial between-country variation in model performance. The SVR model achieved *R*^2^ = 0.978, MAE = 27.4, and RMSE = 44.1 under the random country-year holdout, and *R*^2^ = 0.940, MAE = 44.6, and RMSE = 64.7 under temporal validation. When entire countries were held out using 5-fold grouped cross-validation, pooled out-of-fold performance decreased to *R*^2^ = 0.524, MAE = 158.0, and RMSE = 215.7.

**Conclusion and recommendations:**

The findings of this study indicate that MMR in African countries is influenced by malaria burden and strongly shaped by reproductive, and socioeconomic factors. Reducing maternal mortality in Africa requires the implementation of comprehensive and effective maternal health policy approach that integrates malaria control strategies, malaria-climate surveillance and maternal health literacy programs, alongside strengthening governance, improving institutional capacity, and enhancing health-system performance.

## Introduction

Malaria during pregnancy constitutes a significant health risk to both the mother and the fetus. Despite the presence of pre-existing immunity, adverse maternal outcomes including premature delivery, Fetal Growth Restriction, spontaneous abortion, fetal and maternal mortality may occur (1). WHO’s latest published World malaria report indicated that an estimation of 263 million malaria cases and 597,000 deaths were reported globally in 2023. This reflects an increase of approximately 11 million malaria cases in comparison to the year 2022, while the number of deaths almost remained stable. WHO African Region is accounted for around 95% of malaria deaths; this is the part of the world where many people at risk have restricted access to essential healthcare needed to prevent illnesses, detect and cure disease (2). Furthermore, three main countries in Africa represented more than half of all deaths: Nigeria (31.9%), Congo (11.7%), and Niger (6.1%). Additionally, about 75% of all malaria deaths occurred in children aged under 5 years (3). In WHO African Region, 33 moderates to high transmission countries had an estimation of 35.4 million pregnancies in 2022, of which 12.7 million (36%) were infected with malaria plasmodium and highest estimates were from west and central Africa (4). This persistent and uneven distribution of malaria burden underscores the urgent need to examine the effect of malaria burden in relation to maternal mortality across African countries.

Pregnancy increases susceptibility to malaria infection, particularly among primigravida women. The circulations in placenta and utero-placental are immunologically immature and relatively “malaria-naïve.” This necessitates the growth of an adaptive immune responses within placenta. Repeated exposures to malaria plasmodium during the successive pregnancies will enhance the placenta local immunity responses effectively (5). Published evidence from Congo indicated that malaria infection particularly during the early gestation is association with diminished umbilical artery resistance in late pregnancy with fetal growth restriction in primigravidas. This finding suggests that exposure to infections in early pregnancy may inversely affect the fetal circulation development. It also highlighted the effectiveness of polymerase chain reaction (PCR) test in Plasmodium species identification and improve detection in low-density parasitemia (6). Generally, exposure to Plasmodium falciparum parasite during pregnancy rarely result in fever and therefore remains undetected and untreated in African regions. Thus, nearly one in four pregnancies in African stable transmission regions, have confirmed malaria infection during labor (7).

Based on extensive clinical evidence, malaria during pregnancy leads to adverse maternal and fetal effects. Malaria severely impacts maternal and fetal health, resulting in maternal anemia, fetal growth restriction, preterm birth, maternal and neonatal death. Infants born to mothers with malaria are more susceptible to disease acquisition (8). However, Low Birth Weight ranks among the most commonly documented adverse birth outcomes (9). In the other hand, congenital malaria persists as a significant public health issue specially in high transmission regions, thus strengthening surveillance with targeted interventions are essential to reduce transmissions and improve health outcomes (10). Congenital malaria is associated with nonspecific clinical signs which make it difficult to distinguish from other sepsis (11). Co-infection with HIV further exacerbates parasite density and adverse outcomes. Studies reported that pregnant women with HIV showed higher prevalence and density of malaria parasites (12, 13). Severity and prognosis in malaria pregnant women vary considerably. A recent cohort study reported the association between vital organ dysfunction in severe malaria and the extreme maternal and fetal death, however extreme anemia or hyperparasitemia solely were not necessarily linked to unfavorable clinical outcome (14). These findings highlight the complexity and variation of malaria-related maternal outcomes.

Prevention strategies are well established. WHO recommends the Intermittent preventive treatment in pregnancy (IPTp) with sulfadoxine-pyrimethamine (SP), in moderate to high transmission locations. It is to be initiated in the beginning of second trimester and prescribed at least three times during the entire pregnancy (12). IPTp has been shown significant efficacy in reducing maternal malaria infection and anemia (15, 16). Together with long-lasting insecticidal nets (LLINs), both effectively reduce the risks, but face implementation constraints such as drug resistance and insufficient healthcare access (17). Moreover, climate change has become a key determinant of transmission dynamics. Malaria spread is affected by climatic conditions, land use change and deliberate interventions (18). Evidence showed that climate indicators have inversely impacted malaria control strategies by intensifying the transmission dynamics through favorable rainfall environments and temperature enhanced by socioeconomic exposure processes (19). Temperature proofed to be a significant component of malaria incidence in different locations, because parasite spread is restricted to temperatures between 17 °C and 34 °C (20). Also, A suitable relative humidity of 50–60% prolongs mosquito survival, and rainfall increases vector breeding sites (21). Therefore, effective malaria control necessitates an integrated approaches encompassing not only prevention and treatment with effective antimalarials but also monitoring and control at all levels (22).

Despite extensive clinical evidence assessing the effect of malaria burden at population level, maternal mortality remains unexplored. Most studies focus on individual level or hospital-based estimates, with limited cross-country ecological analyses examining how malaria burden interacts with other determinants to shape population level maternal mortality patterns. To address this gap, the present study employs a machine learning–based ecological framework to investigate the combined effects of malaria burden, reproductive vulnerability, and socioeconomic factors on maternal mortality patterns across malaria-endemic Africa.

## Methods

### Data sources

This ecological study utilized open-access data to predict the Maternal Mortality Ratio (MMR) across 52 African countries from 2000 to 2020. Data was obtained from multiple publicly available sources, including Our World in Data (OWID) (23), World Health Organization (WHO) (24), and NASA Prediction Of Worldwide Energy Resources (POWER) database (25). The primary outcome variable, MMR, was sourced from OWID along with socioeconomic factors such as Gross Domestic Product (GDP per capita and constant USD), Human Development Index (HDI), female labor force participation, fertility rate, and population stratified by female age groups. Educational indicators included female primary, secondary, and tertiary enrollment and completion rates.

Disease burden variables comprised malaria incidence and prevalence, malaria diagnostic confirmation and reporting completeness obtained from WHO, HIV incidence and prevalence among women, and non-communicable diseases including diabetes mellitus. Maternal and perinatal health indicators included stillbirth rate, preterm birth rate and counts, low birthweight prevalence, anemia prevalence in pregnancy and women reproductive age, fertility rate and neonatal health outcomes. Climate indicators were retrieved from NASA POWER and included annual means, minimum, and maximum temperature, relative humidity, daily rainfall, and annual rainfall aggregates. All data sources were harmonized and merged into a single unified country–year panel dataset, stored as a master CSV file. Initially, 54 African countries were considered for data analysis; however, Seychelles and Mauritius were excluded due to extensive data sparsity in the accessed resources, resulting in a final sample of 52 countries. The insufficient data was handled by excluding variables with ≥80% missing data from the analysis such as abortion rates, malaria classifications, and diagnostic variables.

### Data preprocessing

In the preprocessing phase, we evaluated the data type and nature. This included identifying continuous, categorical, temporal, and spatial components variables. Study of the risk factors was performed at this stage. All datasets were standardized to ensure consistency in scale and time period. Outliers were removed and missing data were managed using multivariate model-based imputation. Among the 36 predictor variables retained for modeling, the mean percentage of missing values before imputation was 10.6% (range, 0.0–48.2%); variable-specific missing counts and percentages are provided in (Supplementary Table S5). An Iterative Imputer with a Random Forest Regressor was used to replace missing data. Each variable with missing values was iteratively predicted considering all remaining features until reaching convergence. Median imputation was applied to numeric variables, while one-hot encoding technique was used to transform categorical variables. The target variable, MMR, was transformed using the Yeo–Johnson power to improve normality and enhance the model stability. Pearson correlation coefficients (*r*) were calculated using the final imputed country-year dataset to describe linear associations among the 19 variables displayed in Figure 1. Statistical precision was quantified using a country-cluster percentile bootstrap with 10,000 replicates and a fixed random seed of 42. Entire countries were resampled with replacement so that all 21 annual observations for a selected country remained together within each bootstrap replicate. The 2.5th and 97.5th percentiles of the bootstrap distribution were used as the 95% confidence limits. The intervals are conditional on the completed imputed dataset and do not incorporate additional uncertainty from the imputation procedure.

**Figure 1:**
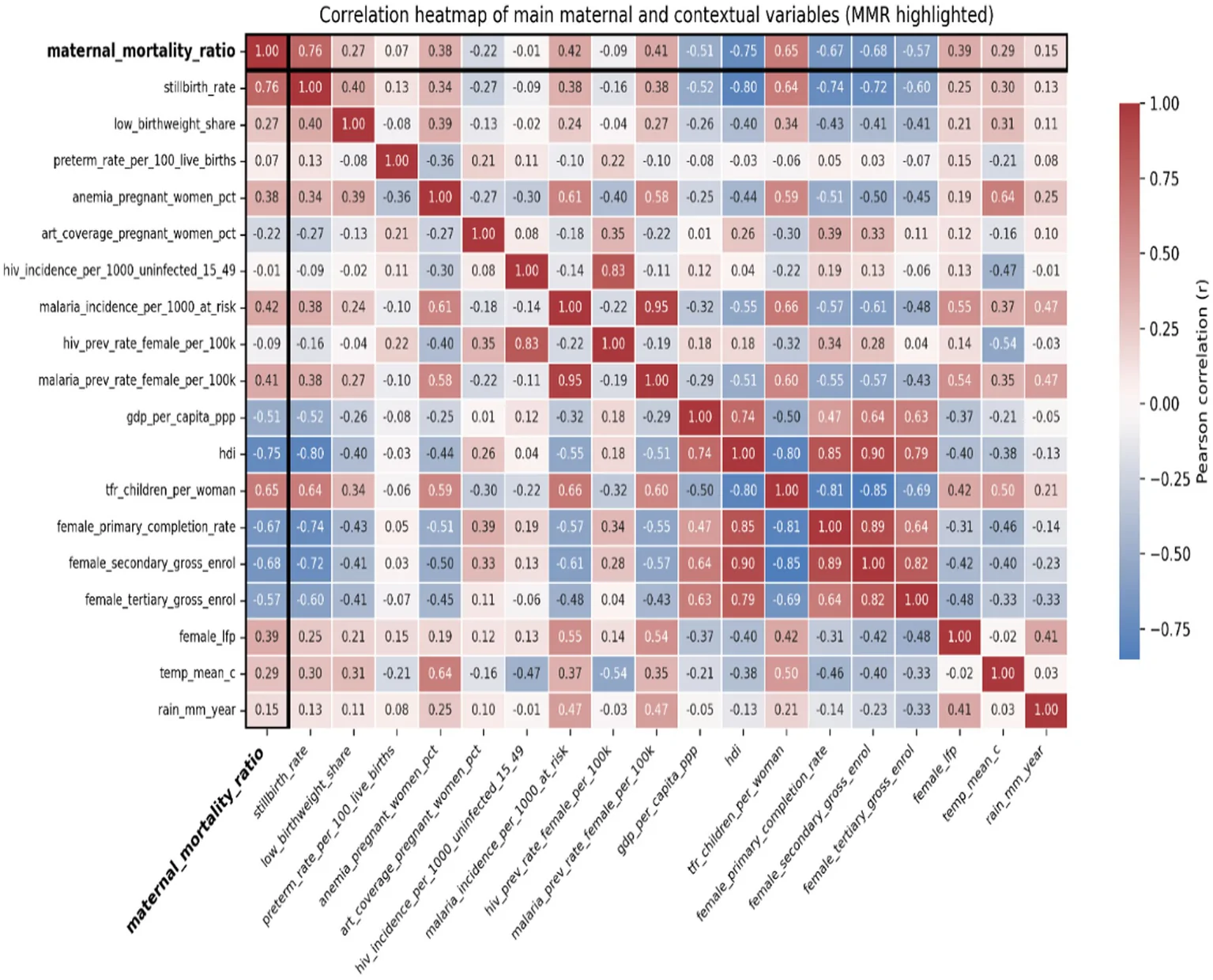
Pearson correlation coefficients heatmap of MMR and contextual variables, based on country-level ecological data in African setting. Cells show pooled Pearson correlation coefficients calculated from 1,092 country-year observations. Corresponding 95% confidence intervals obtained from 10,000 country-cluster bootstrap replicates are reported for all MMR correlations in Supplementary Table S7A and for all pairwise in this figure correlations in Supplementary Table S7B. This figure displays Pearson correlation coefficients (*r*) of MMR and contextual variables. The colors gradient represents the direction and magnitude of association as follow: Light red: Weak to moderate positive correlation. White: No or negligible correlation. Light blue: Weak to moderate negative correlation. *Color scale ranges from −1.0 (perfect negative correlation: deep blue) to +1.0 (perfect positive correlation: deep red). Interpretation: *r*+/−: <0.20 (very weak), 0.20–0.39 (weak), 0.40–0.59 (moderate), 0.60–0.79 (strong), ≥0.80 (very strong).

### Modeling framework

Supervised ML was used to predict MMR across 52 African countries from the selected malaria burden, reproductive, socioeconomic, health, and climatic predictors. The final predictive pipeline median-imputed and standardized numeric predictors and most-frequent-imputed, however one-hot encoded the categorical country variable. Weighted Support Vector Regression (SVR) with a radial basis function kernel was fitted using *C* = 10, gamma = ‘scale’, and epsilon = 0.1. MMR was transformed using a standardized Yeo-Johnson transformation, and observation weights from the W column were applied during model fitting. Principal component analysis (PCA) was not used in either the final random-holdout model or the temporal-validation model; therefore, no PCA components were retained. The initial internal evaluation used a reproducible random split of country-year observations into training (70%; *n* = 764) and test (30%; *n* = 328) sets using a fixed random seed of 42. Because observations from the same country could occur in both subsets, an additional temporal block sensitivity analysis was conducted. In the temporal analysis, observations from 2000 to 2016 were used for model training (*n* = 884), while observations from 2017 to 2020 were reserved for testing (*n* = 208). The same predictor set, preprocessing steps, observation weighting, and SVR hyperparameters were used under both validation schemes. Fifty bootstrap resamples were used under each scheme to calculate mean test-set predictions and 95% bootstrap model intervals (26).

To assess generalization to countries entirely absent from model training, we additionally performed 5-fold grouped cross-validation using country as the grouping variable. All 21 annual observations from each country were assigned to the same fold; therefore, each validation fold contained 10 or 11 countries that were not represented in its corresponding training set. The same no-PCA preprocessing and weighted RBF-SVR settings were used in every fold (*C* = 10, gamma = ‘scale’, epsilon = 0.1, and random seed = 42). Country remained a categorical predictor in the pipeline; previously unseen country categories in a validation fold were handled by the one-hot encoder as unknown categories and encoded as all zeros. Predictions from the five held-out folds were pooled to calculate out-of-fold *R*^2^, MAE, and RMSE across all 1,092 observations.

### Model evaluation

Out-of-sample performance was assessed under three validation schemes: (i) a random 70/30 holdout of country-year observations; (ii) a temporal block holdout in which the model was trained on observations from 2000 to 2016 and evaluated on observations from 2017 to 2020; and (iii) 5-fold GroupKFold cross-validation by country, in which every country was absent from training in exactly one validation fold. Performance was quantified using *R*^2^, MAE, and RMSE. The random and temporal analyses assess prediction within countries represented during training, whereas the grouped-country analysis assesses transfer to countries absent from model training. Comparative performance through models, enhanced final selection, with emphasis on predictive accuracy, and epidemiological plausibility. No formal *a priori* sample size calculation was required. However, sample adequacy was evaluated empirically based on validation stability procedure.

### Geographic visualization of country-level model performance

Country-specific *R*^2^ from the existing per-country model outputs were linked by country name to the Natural Earth 1:10 million administrative level 0 (ADM0) national boundary layer. Each *R*^2^ value was displayed at the national level. No ADM1-level outcome, predictor, prediction, or model-performance estimates were used. Figure 2 therefore represents between-country variation in model performance and does not represent within-country or subnational heterogeneity.

**Figure 2:**
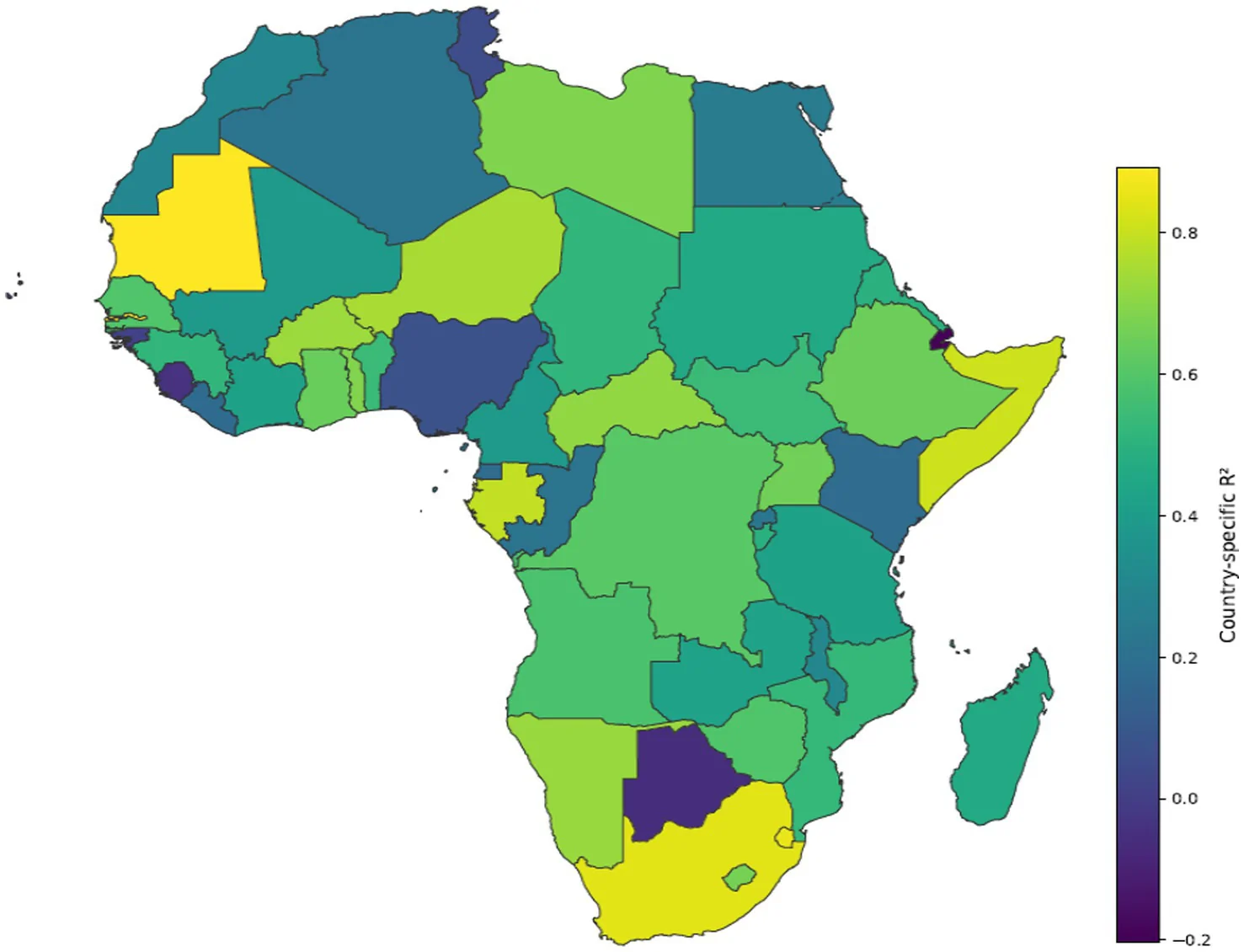
Country level *R*^2^ for MMR (ADM0 level). Country-level model performance for maternal mortality ratio prediction across 52 African countries. Country-specific *R*^2^ estimates from the existing per-country model outputs were linked to Natural Earth 1:10 million administrative level 0 (ADM0) national boundaries. Each colour represents one national estimate. No ADM1-level or other subnational outcome, predictor, prediction, or model-performance data were used; therefore, the map depicts between-country variation in model performance and should not be interpreted as evidence of within-country heterogeneity. Higher *R*^2^ values indicate better country-specific model fit, whereas *R*^2^ values below zero indicate performance below the corresponding mean-only reference model.

### Model explainability

Model-agnostic permutation feature importance was calculated on held-out test data as the decrease in *R*^2^ after permuting one predictor while leaving the remaining predictors unchanged. Each predictor was permuted 10 times using a fixed random seed of 42, and the mean and standard deviation of the resulting decrease in test-set *R*^2^ were recorded. The primary explainability figure uses the temporal holdout model trained on 2000–2016 and evaluated on 2017–2020; the complete temporal and random-holdout importance results are provided in (Supplementary Table S6). Because predictors are correlated, permutation importance was interpreted as model-specific predictive dependence rather than as a causal effect or direction of association.

## Results

Data analysis included Pearson correlation matrices to assess pairwise linear associations and multicollinearity. Pooled country-year coefficients are displayed in Figure 1; country-cluster bootstrap 95% confidence intervals for correlations with MMR and for all variable pairs displayed in Figure 1 are provided in Supplementary Tables S7A,B, respectively.

The results are as follows:

### Correlation heatmap

Figure 1 presents the Pearson correlation heatmap of contextual variables and MMR.

### Malaria burden and MMR

MMR showed moderate positive correlations with malaria incidence (*r* = 0.42; 95% CI, 0.24–0.58) and female malaria prevalence (*r* = 0.41; 95% CI, 0.22–0.58). Both country-cluster bootstrap confidence intervals excluded zero. Malaria incidence was strongly positively correlated with anemia prevalence in pregnant women (*r* = 0.61; 95% CI, 0.44–0.75), while female malaria prevalence was strongly positively correlated with total fertility rate (*r* = 0.60; 95% CI, 0.45–0.72). These pooled ecological associations do not establish causal effects.

### Maternal outcomes and MMR

MMR was strongly positively correlated with stillbirth rate (*r* = 0.76, 95% CI 0.65–0.84) and total fertility rate (*r* = 0.65, 95% CI 0.51–0.76), and moderately correlated with anemia prevalence in pregnant women (*r* = 0.38, 95% CI 0.20–0.55).

### Socioeconomic factors and MMR

MMR showed inverse correlations with HDI (*r* = −0.75; 95% CI, −0.83 to −0.63), female primary completion (*r* = −0.67; 95% CI, −0.79 to −0.52), female secondary enrolment (*r* = −0.68; 95% CI, −0.77 to −0.57), and GDP per capita (*r* = −0.51; 95% CI, −0.64 to −0.40). Malaria incidence and female malaria prevalence were inversely correlated with HDI (*r* = −0.55; 95% CI, −0.69 to −0.37, and *r* = −0.51; 95% CI, −0.65 to −0.35, respectively). Malaria incidence was also inversely correlated with female primary completion (*r* = −0.57; 95% CI, −0.69 to −0.41) and female secondary enrolment (*r* = −0.61; 95% CI, −0.72 to −0.47). All reported intervals excluded zero.

### Climatic indicators and MMR

Mean temperature had a weak positive correlation with MMR (*r* = 0.29, 95% CI 0.04–0.55). The correlation with annual rainfall was weaker and its interval included zero (*r* = 0.15, 95% CI -0.07–0.36).

Overall, the findings indicated that maternal mortality in Africa is moderately associated with malaria burden and strongly structured by fertility and socioeconomic factors. Malaria represented an important, though not isolated, ecological factor of maternal mortality, operating alongside maternal fertility and socioeconomic factors including (GDP, HDI and education attainment).

### Geographic distribution of country-level model performance

Figure 2 presents the geographic distribution of country-specific *R*^2^ values for MMR prediction using ADM0 national boundaries. The *R*^2^ values ranged from −0.202 to 0.891, indicating substantial variation in model performance across the 52 countries. The highest values were observed in Mauritania (*R*^2^ = 0.891), Gambia (*R*^2^ = 0.875), Eswatini (*R*^2^ = 0.855), South Africa (*R*^2^ = 0.842), and Somalia (*R*^2^ = 0.809). Negative values were observed in Djibouti (*R*^2^ = −0.202), Botswana (*R*^2^ = −0.058), Sierra Leone (*R*^2^ = −0.048), and Cape Verde (*R*^2^ = −0.042), indicating performance below the corresponding mean-only reference model in those country-specific evaluations.

All estimates were calculated and mapped at the national level. Figure 2 therefore shows between-country differences in model performance and does not represent ADM1-level predictions, within-country variation, or subnational spatial heterogeneity. The country-specific estimates should be interpreted descriptively because differences may reflect national data characteristics, temporal patterns, unmeasured factors, and country-specific predictor-outcome relationships.

### Model performance under random, temporal, and country-grouped validation

Figure 3 demonstrates the relationship between observed and predicted MMR values in the random 30% country-year test set. Predictions were closely aligned with observations across most of the MMR range, and the model achieved *R*^2^ = 0.978, MAE = 27.4, and RMSE = 44.1. The highest MMR values tended to be underestimated, consistent with regression toward the mean and the relative scarcity of extreme observations.

**Figure 3:**
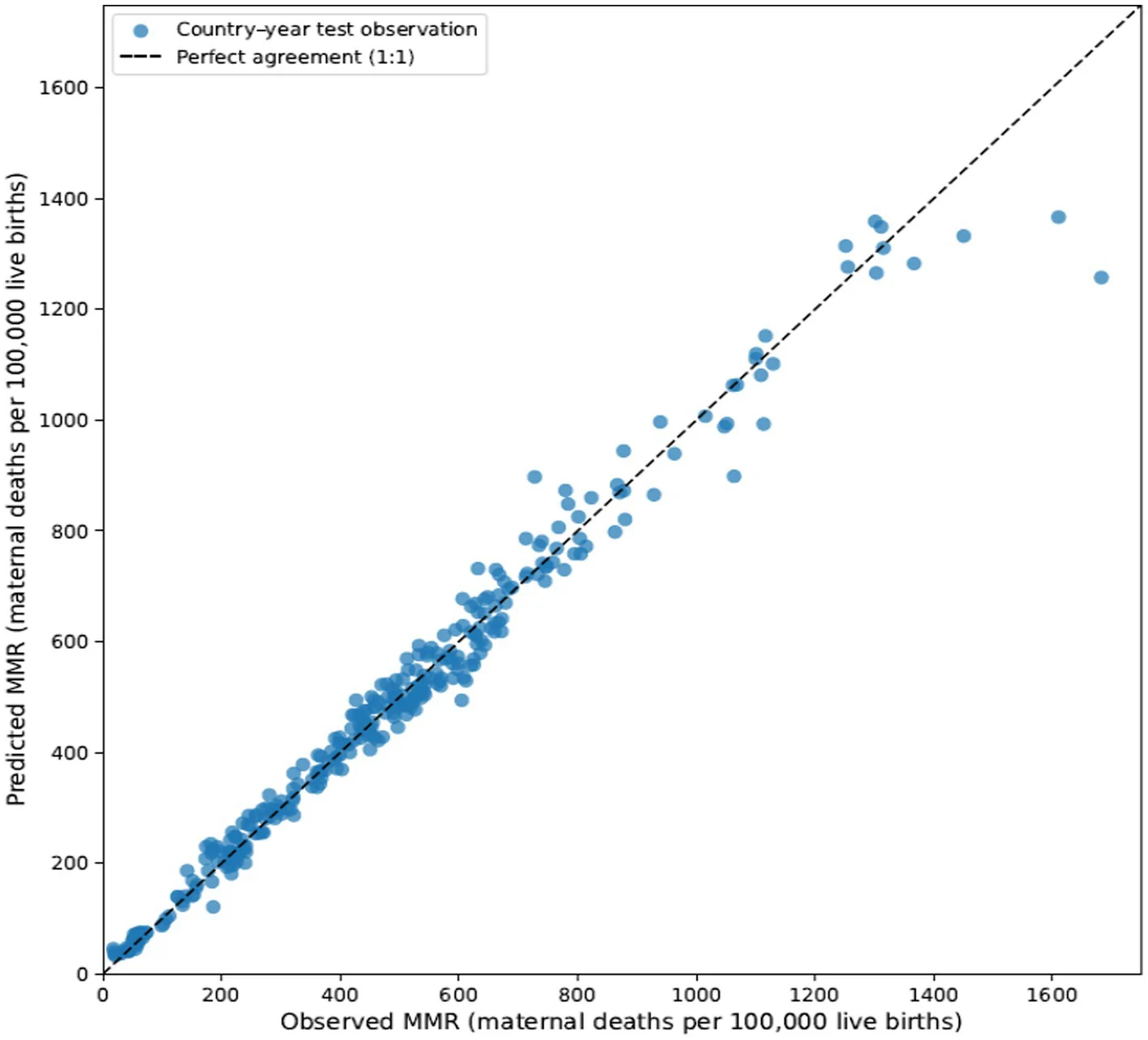
MMR random holdout (Observed_vs_Predicted). Random country-year holdout: observed versus predicted MMR. Each point represents one country-year observation from the random 30% test set. Both axes are expressed as maternal deaths per 100,000 live births, and the dashed diagonal line indicates perfect agreement. Full-test performance was *R*^2^ = 0.978, MAE = 27.4, and RMSE = 44.1; MAE and RMSE are expressed as maternal deaths per 100,000 live births. Because observations from the same countries could occur in both the training and test sets, this is an internal random-holdout result and should be interpreted together with the temporal and country-grouped validation analyses.

To assess the sensitivity of model performance to the longitudinal structure of the data, temporal block sensitivity analysis was performed. The same model configuration was trained on observations from 2000 to 2016 and evaluated on the held-out years 2017–2020. In this temporal test set (*n* = 208), the model achieved *R*^2^ = 0.940, MAE = 44.6, and RMSE = 64.7 (Supplementary Figure S1 and Supplementary Table S1). Performance was lower than under the random country-year split, indicating that the random split provided a more optimistic estimate of predictive performance. Nevertheless, the temporal analysis showed substantial agreement between observed and predicted MMR values for later years, although underestimation was more evident at the highest MMR levels.

Generalization to entirely unseen countries was evaluated using 5-fold grouped cross-validation by country. Across 1,092 pooled out-of-fold predictions from all 52 countries, performance was *R*^2^ = 0.524, MAE = 158.0, and RMSE = 215.7 (Supplementary Figure S2). Mean fold *R*^2^ was 0.464 (SD = 0.217; range, 0.166–0.686), mean fold MAE was 156.8 (SD = 52.9), and mean fold RMSE was 203.3 (SD = 74.4) (Supplementary Table S3). Each country was assigned to one held-out fold, and the complete country-to-fold assignments are provided in (Supplementary Table S4). The marked reduction relative to the random and temporal validations indicates that the original random country-year split substantially overstated performance for countries absent from model training.

Figure 4 displays observed MMR values and bootstrap-mean predictions across the random 30% country-year test set, together with 95% bootstrap model intervals. Observed and predicted values were generally similar, although the intervals were wider for some high-MMR observations. All MMR values are expressed as maternal deaths per 100,000 live births. The *x*-axis is the stored random test-observation index and does not represent temporal or geographic order.

**Figure 4:**
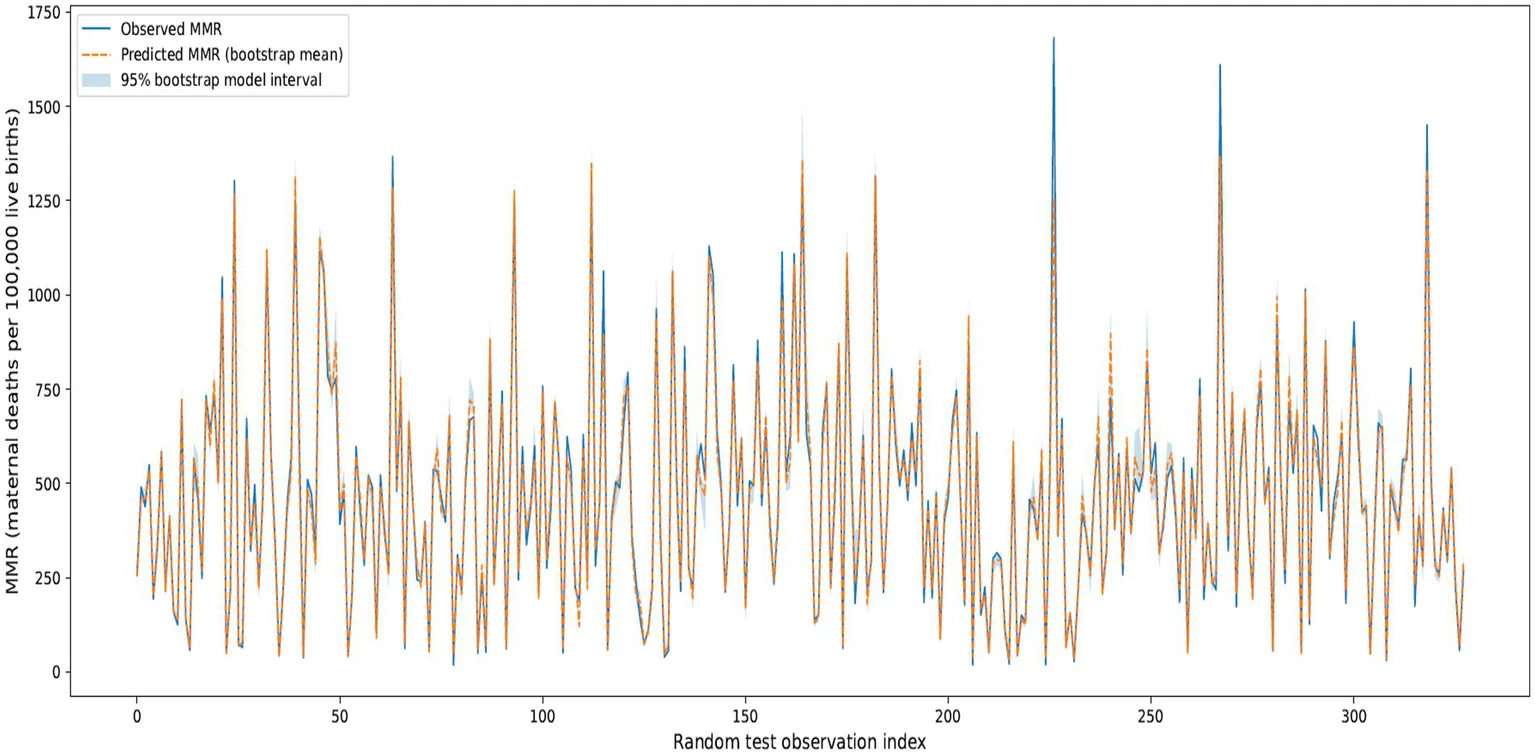
MMR random country-year holdout (Observed and Predicted) with bootstrap interval. Random country-year holdout: observed and predicted MMR across the full random 30% test set (*n* = 328). The *x*-axis is the stored random test-observation index and does not represent time or geographic order; the *y*-axis is expressed as maternal deaths per 100,000 live births. The solid blue line shows observed MMR, the dashed orange line shows the bootstrap-mean prediction, and the shaded area shows the 95% bootstrap model interval from 50 bootstrap refits. Full-test performance was *R*² = 0.978, MAE = 27.4, and RMSE = 44.1; MAE and RMSE are expressed as maternal deaths per 100,000 live births.

Figure 5 provides a zoomed view of test-observation indices 80–140 from the same random 30% country-year holdout. Observed MMR, bootstrap-mean predictions, and 95% bootstrap model intervals are shown in maternal deaths per 100,000 live births. This panel is a visual subset only; the model-performance values reported for the random holdout refer to the complete test set (*n* = 328), not to the 61 observations displayed in the zoomed panel.

**Figure 5:**
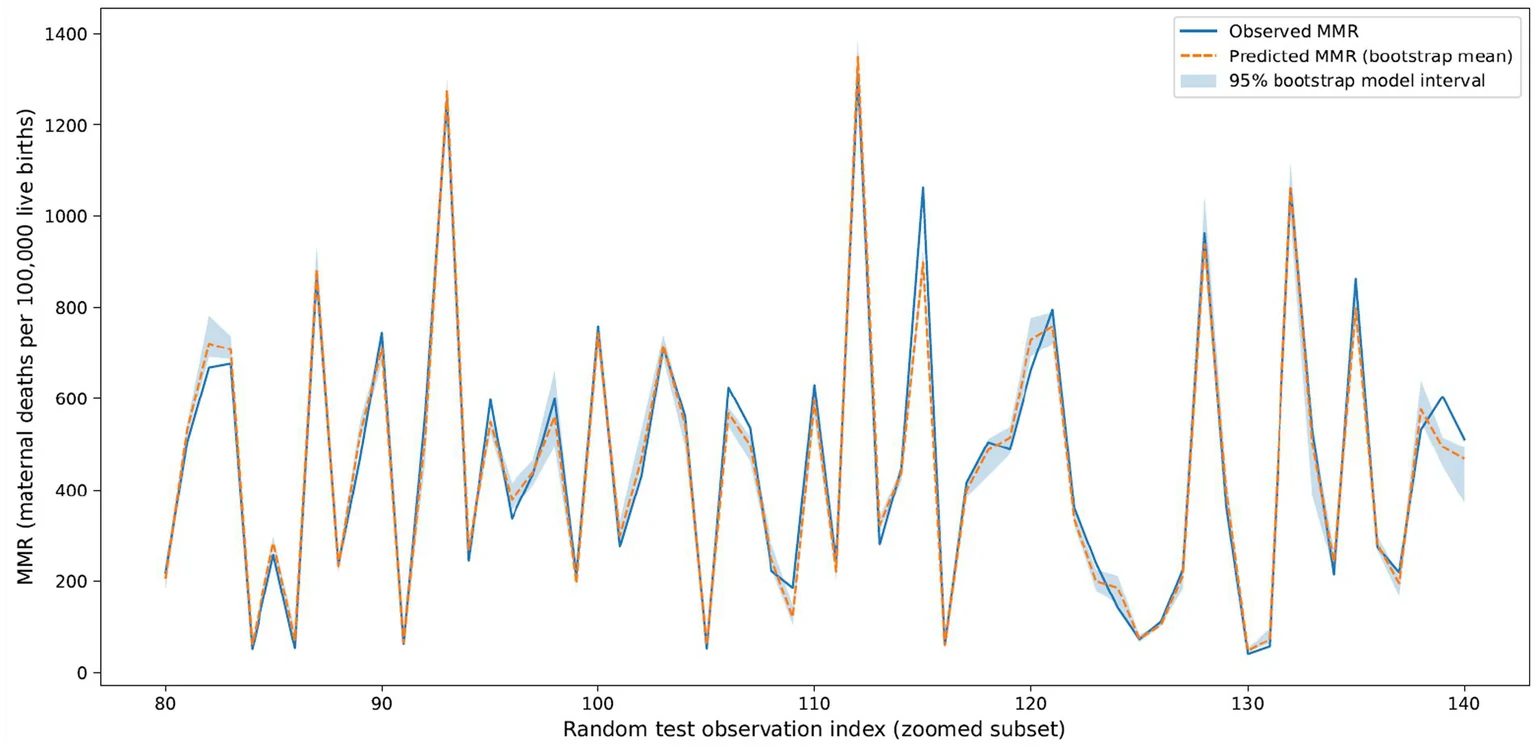
MMR-random country-year holdout (zoomed). The solid blue line represents observed (actual) MMR values. Random country-year holdout: zoomed view of observed and predicted MMR for test observation indices 80–140 (61 observations). The *x*-axis is the stored random test-observation index and does not represent time or geographic order; the *y*-axis is expressed as maternal deaths per 100,000 live births. The solid blue line shows observed MMR, the dashed orange line shows the bootstrap mean prediction, and the shaded area shows the 95% bootstrap model interval. The full-test performance metrics are *R*^2^ = 0.978, MAE = 27.4, and RMSE = 44.1; they were calculated from all 328 random-holdout observations, not from this zoomed subset. MAE and RMSE are expressed as maternal deaths per 100,000 live births.

### Permutation feature importance

In the temporal validation model, the highest permutation importance values were observed for HDI (mean decrease in test-set *R*^2^ = 0.135, SD = 0.017), stillbirth rate (0.134, SD = 0.012), female population aged 15–24 years (0.092, SD = 0.015), preterm-birth count (0.091, SD = 0.017), and total female population (0.091, SD = 0.014) (Figure 6). The country indicator ranked ninth (0.062, SD = 0.003). Female malaria prevalence ranked 29th (0.013, SD = 0.001), and malaria incidence ranked 34th (0.010, SD = 0.001). Thus, malaria indicators contributed to prediction but produced smaller decreases in held-out *R*^2^ than the leading development, reproductive, and population predictors. Complete temporal and random-holdout rankings are reported in Supplementary Table S6.

**Figure 6:**
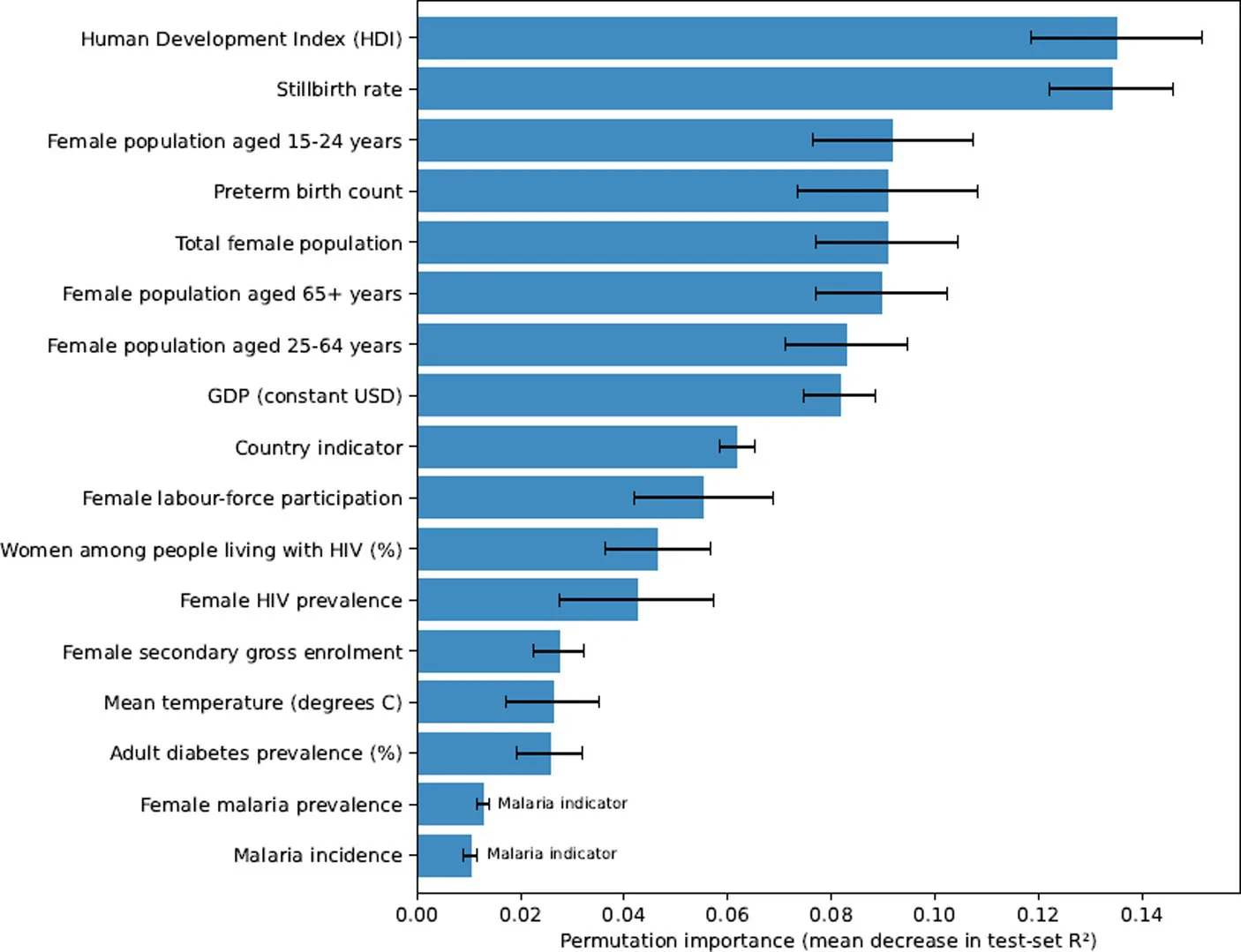
Permutation feature importance. Permutation feature importance in temporal validation of MMR prediction. The SVR model was trained on country-year observations from 2000 to 2016 and evaluated on observations from 2017 to 2020. Importance is the mean decrease in held-out test-set *R*^2^ after permuting one predictor; error bars indicate the standard deviation across 10 permutations. The 15 highest-ranked temporal predictors are shown together with the two prespecified malaria indicators for comparison. Larger values indicate greater model-specific predictive dependence. Values do not indicate causal effects, direction of association, or independent contributions when predictors are correlated.

## Discussion

### Summary of main findings

This analysis shows that MMR in Africa was moderately associated with malaria incidence (*r* = 0.42, 95% CI 0.24–0.58) and female malaria prevalence (*r* = 0.41, 95% CI 0.22–0.58). Stronger associations were observed with stillbirth rate (*r* = 0.76, 95% CI 0.65–0.84), total fertility rate (*r* = 0.65, 95% CI 0.51–0.76), HDI (*r* = −0.75, 95% CI -0.83 to −0.63), and female education indicators. These country-cluster bootstrap intervals indicate the precision of the ecological associations while accounting for repeated annual observations within countries; they do not establish causality and are conditional on the completed imputed dataset. The country-level map showed substantial between-country variation in model performance, with country-specific *R*^2^ values ranging from −0.202 to 0.891. Because all mapped estimates were national, this pattern does not provide evidence of ADM1-level or within-country heterogeneity. The model achieved *R*^2^ = 0.978, MAE = 27.4, and RMSE = 44.1 under the random country-year holdout, and *R*^2^ = 0.940, MAE = 44.6, and RMSE = 64.7 under temporal validation within the same 52 represented countries. In contrast, 5-fold grouped cross-validation that held out entire countries yielded pooled out-of-fold *R*^2^ = 0.524, MAE = 158.0, and RMSE = 215.7. These results show that the model captures substantial within-country temporal structure but transfers less accurately to countries absent from training. The grouped result provides the more relevant estimate of geographic generalization, while the random and temporal results remain useful as internal and later-year sensitivity analyses. All performance metrics describe prediction and do not establish causal effects of the included variables.

The permutation-importance analysis showed that temporal predictive performance depended most strongly on HDI, stillbirth rate, and several population and reproductive indicators. Malaria incidence and female malaria prevalence had positive, non-zero importance but ranked below the leading development and reproductive predictors. This does not contradict their moderate ecological correlations with MMR: correlation describes a pairwise association, whereas permutation importance measures the loss of predictive accuracy when a variable is disrupted in a multivariable model containing correlated predictors. These importance values should therefore support interpretation of the prediction model but should not be used to infer causal priority or intervention effects.

### Interpretation of main findings

The positive association between maternal mortality and malaria burden is consistent with extensive evidence indicating that infection with malaria during pregnancy raises the risks of severe anemia, placental parasitemia, and adverse maternal outcomes (5, 27, 28). Studies demonstrated also that in sub-Saharan Africa, severe pregnancy related anemia has independently been correlated with increased maternal mortality (29). For instance, Hemolytic anemia is common in malarious developing countries. However, pregnant women receiving regular malaria prophylaxis demonstrated anemia improvement (30). This is emphasizing the importance of IPTp during antenatal care. Health literacy and safe programs specifically for young pregnant mothers are also important to mitigate maternal risks (31, 32). Not only the investments on (IPTp) but also on insecticide-treated nets (ITNs), that must be embedded within robust antenatal care platforms to achieve the reduction in adverse maternal outcomes and sustain maternal mortality (33).

The strong negative associations observed with HDI, GDP and female education aligned with the global analyses showing that socioeconomic factors are among the most powerful determinants of maternal survival. Evidence demonstrated significant inverse effect between maternal mortality and GDP (34). In our study, HDI was negatively associated with MMR and anemia. This is consistent with the published evidence reported that the probability of dying from malaria in sub-Saharan countries is inversely related to the HDI (35). Evidence proved that education attainment enhances health literacy, autonomy, uptake of antenatal care, and reported that females with at least secondary education have been shown to be empowered, with high potential to react effectively to maternal issues and navigating healthcare system (36). Evidence from Zambia further demonstrates that educational attainment significantly influences health-seeking behavior (37).

The strong association between MMR and total fertility highlights the structural dimension of reproductive risk. Africa continues to exhibit the highest fertility rates globally, with an average of four children per women throughout their reproductive years, compared to an average of two only globally (38). Early childbearing further complicates the case, as for example in Malawi childbearing starts early with concurrent HIV exposure that elevate maternal morbidity despite antenatal attendance (39).

Country-specific model performance varied across the continent, with high and low *R*^2^ values occurring in multiple African regions. This pattern should be interpreted as variation in model fit between countries rather than as evidence of spatially varying effects within countries. The underlying predictors, outcome, and mapped *R*^2^ estimates were all defined at the national level. Differences in country-specific performance may reflect variation in national data characteristics, temporal patterns, unmeasured determinants, and country-specific predictor-outcome relationships. Figure 2 cannot determine whether these differences were caused by governance, health financing, conflict, institutional capacity, or other contextual factors that were not directly evaluated in the geographic analysis.

The weak but positive associations between climatic variables and MMR likely reflect indirect effects on malaria transmission. Malaria is climate-sensitive, with temperature and rainfall influencing vector and parasite development (40). Empirical evidence from Burundi and other settings confirms that climatic parameters can intensify malaria transmission and undermine control efforts (41). Integrating climate-responsive surveillance into malaria and maternal healthcare planning is a pressing need.

### Recommendations

The findings of this study indicate that maternal mortality in malaria endemic Africa emerges from the interaction between malaria burden, reproductive intensity, and socioeconomic factors. While Malaria contributes to MMR, it represents an important, though not isolated ecological factor, operating alongside maternal fertility and socioeconomic factors. This result further reflects that MMR in Africa is strongly mediated by strengthening governance, improving institutional capacity, and enhancing health-system performance across African countries. These are essential factors for achieving sustainable reductions in maternal mortality. Future analyses using genuine subnational data are needed to guide ADM1-level targeting.

Therefore, strong and effective policies integrating maternal health programs are pivotal across African countries. This comprehensive policy approach includes scaling up Intermittent preventive treatment in pregnancy (IPTp), long-lasting insecticidal nets (LLINs), and indoor residual spraying (IRS). Additionally, malaria and climate response surveillance, and maternal health literacy programs are imperative. However, the effectiveness of this approach depends heavily on sustainable health financing, institutional accountability, coordinating program implementation and strengthening national health-system capacity across African countries. All are critical for translating malaria control investments into measurable maternal survival gains.

## Strengths and limitations

### Strengths

This study has several notable strengths*. First*, this study integrates data across 52 African countries, providing a broad population-level perspective on the relationship between MMR, malaria burden and multiple other factors. *Second*, the use of weighted SVR with a radial basis function kernel enabled nonlinear relationships to be modeled while accounting for observation-level weights, and held-out permutation importance provided a model-agnostic assessment of the predictors contributing to model performance. *Third*, the country-level geographic visualization provides a transparent descriptive comparison of model performance across the 52 African countries. *Fourth*, the use of multi-source datasets spanning two decades (2000–2020), combined with systematic preprocessing and imputation, enhances data comparability and model reliability. *Fifth*, by integrating explanatory and predictive modeling and explicitly linking the study findings to governance and institutional capacity, the study contributes to both global health systems and data informed policy planning.

### Limitations

Few limitations should be considered. *First,* this ecological analysis used country-level aggregates; therefore, the findings are subject to ecological fallacy, and causal inferences at the individual or subnational level cannot be made. Figure 2 displays country-specific *R*^2^ values using ADM0 national boundaries and cannot be used to infer ADM1 level patterns, within-country variation, or subnational heterogeneity. *Second,* variation in data quality, completeness, and measurement across countries may have influenced correlation strength and model performance. *Third,* confounding factors may persist, as important determinants such as health financing levels, conflict exposure, governance quality, and healthcare quality indicators were not included in the analysis and may partly explain unexplained variation. *Finally*, the initial 70/30 split was performed at the country-year observation level, allowing observations from the same country to occur in both training and testing and producing an optimistic estimate of geographic generalization. Temporal validation using 2000–2016 for training and 2017–2020 for testing reduced performance to *R*^2^ = 0.940, MAE = 44.6, and RMSE = 64.7, but all 52 countries remained represented in both periods. When entire countries were held out using 5-fold grouped cross-validation, pooled performance decreased further to *R*^2^ = 0.524, MAE = 158.0, and RMSE = 215.7, with substantial variation across folds. This indicates only moderate and heterogeneous transfer to countries absent from training. The country indicator remained in the pipeline and unseen categories were encoded as all zeros; consequently, the grouped estimates should be interpreted as performance of the specified pipeline rather than as evidence of universal transportability across African settings.

## Conclusion

The findings of this study illustrate that MMR within African countries is influenced by malaria burden but strongly shaped by reproductive, and socioeconomic factors. Reducing maternal deaths in Africa requires implementation of a comprehensive, strong and effective maternal health policy approach. This approach integrates malaria control strategies, malaria surveillance, climate response surveillance, and maternal health literacy programs. Strengthening governance, improving institutional capacity, and enhancing health-system performance across African countries are essential for achieving sustainable reductions in maternal mortality. Future analyses using genuine subnational data are needed to guide ADM1-level targeting.

## Data Availability

The original contributions presented in the study are included in the article/Supplementary material, further inquiries can be directed to the corresponding author/s.

## Glossary

ADM0: Administrative Level 0: National-level administrative boundary (country boundary)
CI: Confidence Intervals
CSV: Comma Separated Values
GDP: Gross Domestic Product
HDI: Human Development Index
HIV: Human Immunodeficiency Virus
IPTp: Intermittent Preventive Treatment in pregnancy
IRS: Indoor Residual Spraying
ITNs: Insecticide Treated Nets
LLINs: Long Lasting Insecticidal Nets
MAE: Mean Absolute Error
ML: Machine Learning
MMR: Maternal Mortality Ratio
NASA: National Aeronautics and Space Administration
OWID: Our World in Data
PCR: Polymerase Chain Reaction
POWER: Prediction Of Worldwide Energy Resources
r: Pearson correlation coefficient
R^2^: Coefficient of Determination
RMSE: Root Mean Squared Error
SD: Standard Deviation
SP: Sulfadoxine-Pyrimethamine
SVR: Support Vector Regression
USD: United States Dollar
WHO: World Health Organization
