# Supplementary material for: Machine learning–based ecological prediction of maternal mortality ratio in malaria-endemic Africa
Source: Front Public Health. 2026 Sep 28;14:1929496. doi: 10.3389/fpubh.2026.1929496 (PMC13655114; doi:10.3389/fpubh.2026.1929496)
Supplement: Supplementary file 1 [file Supplementary_file_1.DOCX]

**Machine Learning–Based Ecological Prediction of Maternal Mortality Ratio in Malaria-Endemic Africa**

**Supplementary Material**

-Supplementary Table S1:

Supplementary Table S1. Comparison of random country-year and temporal holdout performance for maternal mortality ratio ( MMR) prediction.

| **Validation scheme** | **Training data** | **Test data** | **Train n** | **Test n** | **R²** | **MAE** | **RMSE** |
| --- | --- | --- | --- | --- | --- | --- | --- |
| Random country-year holdout | Random 70% of 2000-2020 observations | Random 30% of 2000-2020 observations | 764 | 328 | 0.978 | 27.4 | 44.1 |
| Temporal block holdout | 2000-2016 | 2017-2020 | 884 | 208 | 0.940 | 44.6 | 64.7 |

Supplementary Table S1: R² is dimensionless. MAE and RMSE are expressed as maternal deaths per 100,000 live births. The temporal test set contained four observations for each of the 52 countries and therefore evaluates later-year performance within represented countries rather than performance in entirely unseen countries.

- Supplementary Figure S1:

Supplementary Figure S1.Temporal validation of MMR predictions.

Supplementary Figure S1: Temporal validation of MMR predictions. The SVR model was trained using country-year observations from 2000-2016 and evaluated using observations from 2017-2020 (n = 208). Each point represents one country-year observation. The dashed diagonal line indicates perfect agreement between observed and predicted MMR values. Temporal validation yielded R² = 0.940, MAE = 44.6, and RMSE = 64.7. The highest MMR observations were generally underestimated. Because the same 52 countries were represented in both periods, this analysis assesses performance for later years within represented countries rather than generalization to entirely unseen countries.

- **Supplementary Table S2:**

Supplementary Table S2. Country-specific coefficients of determination used to generate Figure 2

| **Country** | **R²** | **Country** | **R²** |
| --- | --- | --- | --- |
| Algeria | 0.214 | Lesotho | 0.661 |
| Angola | 0.578 | Liberia | 0.178 |
| Benin | 0.540 | Libya | 0.679 |
| Botswana | -0.058 | Madagascar | 0.458 |
| Burkina Faso | 0.725 | Malawi | 0.304 |
| Burundi | 0.491 | Mali | 0.385 |
| Cameroon | 0.387 | Mauritania | 0.891 |
| Cape Verde | -0.042 | Morocco | 0.299 |
| Central African Republic | 0.708 | Mozambique | 0.527 |
| Chad | 0.513 | Namibia | 0.725 |
| Comoros | 0.389 | Niger | 0.750 |
| Congo | 0.215 | Nigeria | 0.072 |
| Cote d Ivoire | 0.421 | Rwanda | 0.228 |
| Democratic Republic of Congo | 0.609 | Sao Tome and Principe | 0.188 |
| Djibouti | -0.202 | Senegal | 0.591 |
| Egypt | 0.251 | Sierra Leone | -0.048 |
| Equatorial Guinea | 0.181 | Somalia | 0.809 |
| Eritrea | 0.491 | South Africa | 0.842 |
| Eswatini | 0.855 | South Sudan | 0.538 |
| Ethiopia | 0.646 | Sudan | 0.452 |
| Gabon | 0.790 | Tanzania | 0.419 |
| Gambia | 0.875 | Togo | 0.684 |
| Ghana | 0.639 | Tunisia | 0.049 |
| Guinea | 0.515 | Uganda | 0.655 |
| Guinea Bissau | 0.010 | Zambia | 0.430 |
| Kenya | 0.180 | Zimbabwe | 0.592 |

Supplementary Table S2: Country-specific R² estimates were mapped using Natural Earth administrative level 0 national boundaries. No ADM1-level or other subnational estimates were generated. Values below zero indicate performance below the corresponding mean-only reference model.

-Supplementary Figure S2:

Supplementary Figure S2. Grouped Country Cross Validation _scatter

Supplementary Figure S2: Observed versus out-of-fold predicted maternal mortality ratio under 5-fold grouped cross-validation by country. All 21 annual observations from each country were held out together, and each of the 52 countries was absent from training in one-fold. The plot includes all 1,092 pooled out-of-fold predictions. The dashed diagonal line indicates perfect agreement. Pooled performance was R² = 0.524, MAE = 158.0, and RMSE = 215.7; MAE and RMSE are expressed as maternal deaths per 100,000 live births.

**- Supplementary Table S3:**

Supplementary Table S3**.** Fold-specific performance of 5-fold grouped cross-validation by country for maternal mortality ratio prediction.

| **Fold** | **Train countries** | **Test countries** | **Train n** | **Test n** | **R²** | **MAE** | **RMSE** |
| --- | --- | --- | --- | --- | --- | --- | --- |
| 1 | 41 | 11 | 861 | 231 | 0.666 | 135.9 | 165.2 |
| 2 | 41 | 11 | 861 | 231 | 0.166 | 240.8 | 322.6 |
| 3 | 42 | 10 | 882 | 210 | 0.418 | 175.3 | 225.2 |
| 4 | 42 | 10 | 882 | 210 | 0.385 | 116.4 | 133.8 |
| 5 | 42 | 10 | 882 | 210 | 0.686 | 115.4 | 169.6 |

Supplementary Table S3: All observations from a country were assigned to one held-out fold. R² is dimensionless; MAE and RMSE are expressed as maternal deaths per 100,000 live births. Pooled out-of-fold performance across all 1,092 observations was R² = 0.524, MAE = 158.0, and RMSE = 215.7. The categorical country predictor was retained; country categories absent from a training fold were handled as unknown and encoded as all zeros in the corresponding validation fold.

**-Supplementary Table S4:**

Supplementary Table S4. Country assignments used for 5-fold grouped cross-validation.

| **Country** | **Fold** | **Country** | **Fold** |
| --- | --- | --- | --- |
| Burundi | 1 | Guinea | 3 |
| Comoros | 1 | Lesotho | 3 |
| Egypt | 1 | Mali | 3 |
| Gabon | 1 | Niger | 3 |
| Guinea Bissau | 1 | Sierra Leone | 3 |
| Liberia | 1 | Tanzania | 3 |
| Libya | 1 | Benin | 4 |
| Morocco | 1 | Cape Verde | 4 |
| South Africa | 1 | Cote d Ivoire | 4 |
| Tunisia | 1 | Eritrea | 4 |
| Zimbabwe | 1 | Ghana | 4 |
| Algeria | 2 | Malawi | 4 |
| Burkina Faso | 2 | Namibia | 4 |
| Chad | 2 | Sao Tome and Principe | 4 |
| Djibouti | 2 | Senegal | 4 |
| Ethiopia | 2 | Sudan | 4 |
| Kenya | 2 | Angola | 5 |
| Mauritania | 2 | Cameroon | 5 |
| Nigeria | 2 | Congo | 5 |
| Somalia | 2 | Equatorial Guinea | 5 |
| Togo | 2 | Gambia | 5 |
| Zambia | 2 | Madagascar | 5 |
| Botswana | 3 | Mozambique | 5 |
| Central African Republic | 3 | Rwanda | 5 |
| Democratic Republic of Congo | 3 | South Sudan | 5 |
| Eswatini | 3 | Uganda | 5 |

Supplementary Table S4: Each of the 52 countries appears in one and only one held-out fold; all 21 annual observations for that country were retained together.

**-Supplementary Table S5:**

Supplementary Table S5. Missing values in retained predictor variables before imputation.

| **Predictor** | **Analysis field** | **Missing n** | **Total n** | **Missing %** |
| --- | --- | --- | --- | --- |
| Female tertiary gross enrolment | female_tertiary_gross_enrol | 526 | 1092 | 48.2 |
| Preterm births (number) | preterm_births_number | 520 | 1092 | 47.6 |
| Preterm-birth rate per 100 live births | preterm_rate_per_100_live_births | 520 | 1092 | 47.6 |
| Female secondary gross enrolment | female_secondary_gross_enrol | 495 | 1092 | 45.3 |
| Female primary completion rate | female_primary_completion_rate | 409 | 1092 | 37.5 |
| Adult diabetes prevalence (%) | diabetes_prev_adults_pct | 342 | 1092 | 31.3 |
| Low-birthweight share | low_birthweight_share | 336 | 1092 | 30.8 |
| Female primary gross enrolment | female_primary_gross_enrol | 262 | 1092 | 24.0 |
| ART coverage among pregnant women (%) | art_coverage_pregnant_women_pct | 112 | 1092 | 10.3 |
| Female disease prevalence: DS (per 100,000) | ds_prev_rate_female_per_100k | 63 | 1092 | 5.8 |
| HIV incidence (per 1,000 uninfected aged 15-49) | hiv_incidence_per_1000_uninfected_15_49 | 63 | 1092 | 5.8 |
| Female HIV prevalence (per 100,000) | hiv_prev_rate_female_per_100k | 63 | 1092 | 5.8 |
| Female IBD prevalence (per 100,000) | ibd_prev_rate_female_per_100k | 63 | 1092 | 5.8 |
| Malaria incidence (per 1,000 at risk) | malaria_incidence_per_1000_at_risk | 63 | 1092 | 5.8 |
| Female malaria prevalence (per 100,000) | malaria_prev_rate_female_per_100k | 63 | 1092 | 5.8 |
| Female MS prevalence (per 100,000) | ms_prev_rate_female_per_100k | 63 | 1092 | 5.8 |
| Female RA prevalence (per 100,000) | ra_prev_rate_female_per_100k | 63 | 1092 | 5.8 |
| Human Development Index (HDI) | hdi | 60 | 1092 | 5.5 |
| GDP per capita (PPP) | gdp_per_capita_ppp | 55 | 1092 | 5.0 |
| GDP (constant USD) | gdp_constant_usd | 35 | 1092 | 3.2 |
| Anaemia prevalence in pregnant women (%) | anemia_pregnant_women_pct | 0 | 1092 | 0.0 |
| Anaemia in women of reproductive age 15-29 | anemia_wra_15_29 | 0 | 1092 | 0.0 |
| Female labour-force participation | female_lfp | 0 | 1092 | 0.0 |
| Female population aged 15-24 years | female_pop_15_24 | 0 | 1092 | 0.0 |
| Female population aged 25-64 years | female_pop_25_64 | 0 | 1092 | 0.0 |
| Female population aged 65+ years | female_pop_65_plus | 0 | 1092 | 0.0 |
| Total female population | female_pop_total | 0 | 1092 | 0.0 |
| Daily rainfall (mm/day) | rain_mm_per_day | 0 | 1092 | 0.0 |
| Annual rainfall (mm/year) | rain_mm_year | 0 | 1092 | 0.0 |
| Relative humidity (%) | rel_humidity_pct | 0 | 1092 | 0.0 |
| Women among people living with HIV (%) | share_women_among_plhiv_pct | 0 | 1092 | 0.0 |
| Stillbirth rate | stillbirth_rate | 0 | 1092 | 0.0 |
| Maximum temperature (degrees C) | temp_max_c | 0 | 1092 | 0.0 |
| Mean temperature (degrees C) | temp_mean_c | 0 | 1092 | 0.0 |
| Minimum temperature (degrees C) | temp_min_c | 0 | 1092 | 0.0 |
| Total fertility rate (children per woman) | tfr_children_per_woman | 0 | 1092 | 0.0 |

Supplementary Table S5: Missingness was calculated before imputation across 1,092 country-year observations. The 36 retained predictors contained 4,176 missing values among 39,312 cells; mean variable-level missingness was 10.6% (range, 0.0%-48.2%), and 20 predictors contained at least one missing value. Variables excluded before modeling because they had at least 80% missingness are not included. Administrative identifiers, year, the MMR target, and the observation-weight column were not predictor variables subject to this imputation summary.

- **Supplementary Table S6**:

Supplementary Table S6. Permutation feature importance under temporal and random country-year validation.

| **feature** | **display_name** | **temporal_rank** | **temporal_importance_mean** | **temporal_importance_std** | **random_rank** | **random_importance_mean** | **random_importance_std** |
| --- | --- | --- | --- | --- | --- | --- | --- |
| hdi | Human Development Index (HDI) | 1 | 0.134952897 | 0.016523974 | 2 | 0.16611727 | 0.016338151 |
| stillbirth_rate | Stillbirth rate | 2 | 0.133967317 | 0.011934159 | 1 | 0.244363735 | 0.02310286 |
| female_pop_15_24 | Female population aged 15-24 years | 3 | 0.091781139 | 0.015440094 | 8 | 0.049365732 | 0.008915964 |
| preterm_births_number | Preterm birth count | 4 | 0.090841404 | 0.017465766 | 7 | 0.050433422 | 0.010533443 |
| female_pop_total | Total female population | 5 | 0.090671607 | 0.013724478 | 12 | 0.047312122 | 0.008513215 |
| female_pop_65_plus | Female population aged 65+ years | 6 | 0.089637882 | 0.012684138 | 5 | 0.06195445 | 0.009220739 |
| female_pop_25_64 | Female population aged 25-64 years | 7 | 0.082825969 | 0.011707533 | 13 | 0.043862805 | 0.008210059 |
| gdp_constant_usd | GDP (constant USD) | 8 | 0.081527214 | 0.006843619 | 14 | 0.037252047 | 0.009148309 |
| country | Country indicator | 9 | 0.061758105 | 0.003474418 | 9 | 0.048825447 | 0.00382843 |
| female_lfp | Female labour-force participation | 10 | 0.055248255 | 0.013347366 | 11 | 0.047548764 | 0.005592626 |
| share_women_among_plhiv_pct | Women among people living with HIV (%) | 11 | 0.046393648 | 0.010190914 | 6 | 0.05278703 | 0.011058937 |
| hiv_prev_rate_female_per_100k | Female HIV prevalence | 12 | 0.042447621 | 0.014899941 | 10 | 0.047797619 | 0.009572675 |
| female_secondary_gross_enrol | Female secondary gross enrolment | 13 | 0.027399935 | 0.004931977 | 18 | 0.023653602 | 0.00475644 |
| temp_mean_c | Mean temperature (degrees C) | 14 | 0.02622075 | 0.009057629 | 19 | 0.021459932 | 0.004959908 |
| diabetes_prev_adults_pct | Adult diabetes prevalence (%) | 15 | 0.025652951 | 0.006260792 | 17 | 0.0315515 | 0.0081763 |
| temp_max_c | Temp max c | 16 | 0.024789549 | 0.004427246 | 30 | 0.011151778 | 0.003702323 |
| preterm_rate_per_100_live_births | Preterm rate per 100 live births | 17 | 0.02422666 | 0.006418584 | 34 | 0.009695814 | 0.002817407 |
| tfr_children_per_woman | Total fertility rate | 18 | 0.024216753 | 0.005517068 | 4 | 0.065785955 | 0.009431139 |
| low_birthweight_share | Low-birthweight share | 19 | 0.02222235 | 0.006288493 | 3 | 0.077491068 | 0.010541183 |
| ibd_prev_rate_female_per_100k | Ibd prev rate female per 100k | 20 | 0.018706071 | 0.007010899 | 31 | 0.011073891 | 0.00355859 |
| female_tertiary_gross_enrol | Female tertiary gross enrol | 21 | 0.018216673 | 0.006682458 | 24 | 0.01719596 | 0.004774409 |
| rel_humidity_pct | Rel humidity pct | 22 | 0.016541814 | 0.001491072 | 29 | 0.012116675 | 0.001741305 |
| ms_prev_rate_female_per_100k | Ms prev rate female per 100k | 23 | 0.016229529 | 0.008605706 | 36 | 0.008113861 | 0.003321612 |
| rain_mm_per_day | Rain mm per day | 24 | 0.016192638 | 0.002459275 | 37 | 0.006239286 | 0.00128512 |
| female_primary_completion_rate | Female primary completion rate | 25 | 0.016093541 | 0.003890541 | 22 | 0.019369314 | 0.003954757 |
| ds_prev_rate_female_per_100k | Ds prev rate female per 100k | 26 | 0.015635315 | 0.002409203 | 16 | 0.031844073 | 0.004370299 |
| rain_mm_year | Rain mm year | 27 | 0.015399848 | 0.002290961 | 38 | 0.006046999 | 0.001494217 |
| temp_min_c | Temp min c | 28 | 0.013616781 | 0.004122133 | 28 | 0.013864129 | 0.003407738 |
| malaria_prev_rate_female_per_100k | Female malaria prevalence | 29 | 0.012742813 | 0.001079295 | 23 | 0.0175933 | 0.00240304 |
| ra_prev_rate_female_per_100k | Ra prev rate female per 100k | 30 | 0.012613095 | 0.004828842 | 25 | 0.016806483 | 0.009755553 |
| art_coverage_pregnant_women_pct | Art coverage pregnant women pct | 31 | 0.012428389 | 0.004602024 | 33 | 0.010812835 | 0.002868741 |
| female_primary_gross_enrol | Female primary gross enrol | 32 | 0.011659168 | 0.003671796 | 32 | 0.011043738 | 0.002770247 |
| gdp_per_capita_ppp | GDP per capita (PPP) | 33 | 0.010710539 | 0.003476526 | 21 | 0.020339445 | 0.007004656 |
| malaria_incidence_per_1000_at_risk | Malaria incidence | 34 | 0.01036028 | 0.001321135 | 26 | 0.014276398 | 0.001961126 |
| anemia_pregnant_women_pct | Anaemia in pregnant women (%) | 35 | 0.009811786 | 0.002088587 | 35 | 0.008885218 | 0.001451701 |
| anemia_wra_15_29 | Anemia wra 15 29 | 36 | 0.009257056 | 0.002179449 | 27 | 0.014127102 | 0.002892527 |
| hiv_incidence_per_1000_uninfected_15_49 | Hiv incidence per 1000 uninfected 15 49 | 37 | 0.009022134 | 0.004077403 | 15 | 0.034175064 | 0.007825023 |
| year | Year | 38 | 0.000245602 | 0.001441869 | 20 | 0.021358546 | 0.00286265 |

Supplementary Table S6: Importance is the mean decrease in held-out test-set R² after permuting one predictor, with standard deviation across 10 permutations. Temporal importance used the model trained on 2000-2016 and tested on 2017-2020; random-holdout importance used the final 70/30 country-year split. Larger values indicate greater model-specific predictive dependence. Rankings are affected by correlation among predictors and do not represent causal effects or direction of association.

**-Supplementary Table S7A**:

Supplementary Table S7A . Pearson correlations between maternal mortality ratio and Figure 1 predictors with 95% country-cluster bootstrap confidence intervals.

| **Predictor** | **Pearson r** | **95% CI** | **CI excludes zero** |
| --- | --- | --- | --- |
| Stillbirth rate | 0.76 | 0.65 to 0.84 | Yes |
| Low-birthweight share | 0.27 | 0.05 to 0.47 | Yes |
| Preterm-birth rate | 0.07 | -0.10 to 0.26 | No |
| Anaemia prevalence in pregnant women | 0.38 | 0.20 to 0.55 | Yes |
| ART coverage among pregnant women | -0.22 | -0.36 to -0.10 | Yes |
| HIV incidence | -0.01 | -0.20 to 0.16 | No |
| Malaria incidence | 0.42 | 0.24 to 0.58 | Yes |
| Female HIV prevalence | -0.09 | -0.29 to 0.13 | No |
| Female malaria prevalence | 0.41 | 0.22 to 0.58 | Yes |
| GDP per capita (PPP) | -0.51 | -0.64 to -0.40 | Yes |
| Human Development Index | -0.75 | -0.83 to -0.63 | Yes |
| Total fertility rate | 0.65 | 0.51 to 0.76 | Yes |
| Female primary completion rate | -0.67 | -0.79 to -0.52 | Yes |
| Female secondary gross enrolment | -0.68 | -0.77 to -0.57 | Yes |
| Female tertiary gross enrolment | -0.57 | -0.66 to -0.48 | Yes |
| Female labour-force participation | 0.39 | 0.18 to 0.57 | Yes |
| Mean temperature | 0.29 | 0.04 to 0.55 | Yes |
| Annual rainfall | 0.15 | -0.07 to 0.36 | No |

Supplementary Table S7A : Pearson coefficients were calculated from 1,092 observations representing 52 countries with 21 annual observations per country. Confidence limits are the 2.5th and 97.5th percentiles from 10,000 bootstrap replicates in which countries, rather than individual country-year rows, were sampled with replacement (random seed = 42). An interval excluding zero indicates that the bootstrap interval did not cross zero; no row-level p-values were calculated. Intervals are conditional on the completed imputed dataset and do not propagate imputation uncertainty.

**-Supplementary Table S7B:**

Supplementary Table S7B. Pairwise Pearson correlations among all variables displayed in Figure 1 with 95% country-cluster bootstrap confidence intervals.

| **variable_1** | **pearson_r** | **cluster_bootstrap_ci_lower** | **cluster_bootstrap_ci_upper** |
| --- | --- | --- | --- |
| maternal_mortality_ratio | 0.758549 | 0.648542 | 0.84496 |
| maternal_mortality_ratio | 0.268788 | 0.0504 | 0.465439 |
| maternal_mortality_ratio | 0.073761 | -0.10448 | 0.263839 |
| maternal_mortality_ratio | 0.382384 | 0.204315 | 0.554527 |
| maternal_mortality_ratio | -0.22439 | -0.35533 | -0.09614 |
| maternal_mortality_ratio | -0.00711 | -0.20036 | 0.160936 |
| maternal_mortality_ratio | 0.415007 | 0.23581 | 0.575798 |
| maternal_mortality_ratio | -0.0852 | -0.29351 | 0.129725 |
| maternal_mortality_ratio | 0.408987 | 0.222991 | 0.5811 |
| maternal_mortality_ratio | -0.50951 | -0.63609 | -0.40201 |
| maternal_mortality_ratio | -0.74735 | -0.83462 | -0.63438 |
| maternal_mortality_ratio | 0.645994 | 0.507294 | 0.761048 |
| maternal_mortality_ratio | -0.67185 | -0.78936 | -0.51798 |
| maternal_mortality_ratio | -0.67988 | -0.76642 | -0.57106 |
| maternal_mortality_ratio | -0.57203 | -0.66106 | -0.47763 |
| maternal_mortality_ratio | 0.392922 | 0.178367 | 0.573502 |
| maternal_mortality_ratio | 0.291341 | 0.043575 | 0.552911 |
| maternal_mortality_ratio | 0.154377 | -0.06963 | 0.363016 |
| stillbirth_rate | 0.403171 | 0.15892 | 0.609183 |
| stillbirth_rate | 0.130672 | -0.11089 | 0.376947 |
| stillbirth_rate | 0.341501 | 0.106469 | 0.558509 |
| stillbirth_rate | -0.27426 | -0.40968 | -0.153 |
| stillbirth_rate | -0.08781 | -0.28407 | 0.152302 |
| stillbirth_rate | 0.38479 | 0.177821 | 0.591243 |
| stillbirth_rate | -0.15806 | -0.36735 | 0.076137 |
| stillbirth_rate | 0.379588 | 0.17454 | 0.59115 |
| stillbirth_rate | -0.52358 | -0.6876 | -0.38388 |
| stillbirth_rate | -0.80007 | -0.86429 | -0.72243 |
| stillbirth_rate | 0.644766 | 0.470935 | 0.78891 |
| stillbirth_rate | -0.73528 | -0.82099 | -0.63029 |
| stillbirth_rate | -0.72353 | -0.80599 | -0.62303 |
| stillbirth_rate | -0.59695 | -0.69076 | -0.48646 |
| stillbirth_rate | 0.251976 | -0.04121 | 0.518005 |
| stillbirth_rate | 0.303845 | 0.052135 | 0.593248 |
| stillbirth_rate | 0.130004 | -0.10715 | 0.344573 |
| low_birthweight_share | -0.07582 | -0.28024 | 0.111511 |
| low_birthweight_share | 0.391657 | 0.152557 | 0.601654 |
| low_birthweight_share | -0.12835 | -0.28409 | 0.02581 |
| low_birthweight_share | -0.02123 | -0.22676 | 0.217728 |
| low_birthweight_share | 0.238228 | -0.01406 | 0.473307 |
| low_birthweight_share | -0.0359 | -0.26989 | 0.193249 |
| low_birthweight_share | 0.269653 | 0.034087 | 0.488737 |
| low_birthweight_share | -0.26469 | -0.50239 | -0.04774 |
| low_birthweight_share | -0.40336 | -0.59645 | -0.17213 |
| low_birthweight_share | 0.338477 | 0.118763 | 0.534559 |
| low_birthweight_share | -0.42994 | -0.59711 | -0.24471 |
| low_birthweight_share | -0.40705 | -0.60422 | -0.17808 |
| low_birthweight_share | -0.41395 | -0.59425 | -0.1198 |
| low_birthweight_share | 0.21054 | -0.09489 | 0.478739 |
| low_birthweight_share | 0.313166 | 0.126137 | 0.55026 |
| low_birthweight_share | 0.109699 | -0.14796 | 0.329344 |
| preterm_rate_per_100_live_births | -0.36291 | -0.54937 | -0.12004 |
| preterm_rate_per_100_live_births | 0.213447 | 0.054652 | 0.356366 |
| preterm_rate_per_100_live_births | 0.105306 | -0.06897 | 0.312528 |
| preterm_rate_per_100_live_births | -0.1047 | -0.30573 | 0.110203 |
| preterm_rate_per_100_live_births | 0.216337 | -0.02499 | 0.458971 |
| preterm_rate_per_100_live_births | -0.10255 | -0.28883 | 0.103644 |
| preterm_rate_per_100_live_births | -0.08092 | -0.36363 | 0.212492 |
| preterm_rate_per_100_live_births | -0.03049 | -0.28188 | 0.235291 |
| preterm_rate_per_100_live_births | -0.0554 | -0.30623 | 0.203829 |
| preterm_rate_per_100_live_births | 0.052206 | -0.16015 | 0.252957 |
| preterm_rate_per_100_live_births | 0.028911 | -0.21952 | 0.298218 |
| preterm_rate_per_100_live_births | -0.06943 | -0.24121 | 0.160099 |
| preterm_rate_per_100_live_births | 0.154182 | -0.00348 | 0.309452 |
| preterm_rate_per_100_live_births | -0.21183 | -0.4255 | -0.01595 |
| preterm_rate_per_100_live_births | 0.080813 | -0.10324 | 0.268321 |
| anemia_pregnant_women_pct | -0.26547 | -0.39532 | -0.12391 |
| anemia_pregnant_women_pct | -0.29647 | -0.45658 | -0.07834 |
| anemia_pregnant_women_pct | 0.608692 | 0.43862 | 0.751131 |
| anemia_pregnant_women_pct | -0.39782 | -0.57662 | -0.17685 |
| anemia_pregnant_women_pct | 0.580606 | 0.404935 | 0.729109 |
| anemia_pregnant_women_pct | -0.2461 | -0.53488 | -0.00343 |
| anemia_pregnant_women_pct | -0.4411 | -0.62925 | -0.22039 |
| anemia_pregnant_women_pct | 0.588706 | 0.433027 | 0.725192 |
| anemia_pregnant_women_pct | -0.50634 | -0.66332 | -0.32764 |
| anemia_pregnant_women_pct | -0.50022 | -0.66591 | -0.3045 |
| anemia_pregnant_women_pct | -0.45433 | -0.59028 | -0.30872 |
| anemia_pregnant_women_pct | 0.188947 | -0.037 | 0.397611 |
| anemia_pregnant_women_pct | 0.636888 | 0.522254 | 0.774699 |
| anemia_pregnant_women_pct | 0.253054 | 0.045639 | 0.448852 |
| art_coverage_pregnant_women_pct | 0.082304 | -0.01855 | 0.161196 |
| art_coverage_pregnant_women_pct | -0.17501 | -0.3043 | -0.04545 |
| art_coverage_pregnant_women_pct | 0.353252 | 0.24172 | 0.453034 |
| art_coverage_pregnant_women_pct | -0.22354 | -0.34001 | -0.10887 |
| art_coverage_pregnant_women_pct | 0.012253 | -0.12546 | 0.198131 |
| art_coverage_pregnant_women_pct | 0.264564 | 0.11664 | 0.43918 |
| art_coverage_pregnant_women_pct | -0.30001 | -0.45107 | -0.15407 |
| art_coverage_pregnant_women_pct | 0.390622 | 0.270434 | 0.511936 |
| art_coverage_pregnant_women_pct | 0.329816 | 0.168365 | 0.498805 |
| art_coverage_pregnant_women_pct | 0.108963 | -0.04971 | 0.368978 |
| art_coverage_pregnant_women_pct | 0.123336 | -0.01994 | 0.253059 |
| art_coverage_pregnant_women_pct | -0.16221 | -0.28152 | -0.01821 |
| art_coverage_pregnant_women_pct | 0.095334 | -0.04587 | 0.23611 |
| hiv_incidence_per_1000_uninfected_15_49 | -0.13602 | -0.33372 | 0.175557 |
| hiv_incidence_per_1000_uninfected_15_49 | 0.832264 | 0.809346 | 0.862338 |
| hiv_incidence_per_1000_uninfected_15_49 | -0.11459 | -0.2922 | 0.169105 |
| hiv_incidence_per_1000_uninfected_15_49 | 0.123965 | -0.09101 | 0.335822 |
| hiv_incidence_per_1000_uninfected_15_49 | 0.041865 | -0.14573 | 0.225299 |
| hiv_incidence_per_1000_uninfected_15_49 | -0.21551 | -0.40096 | 0.024701 |
| hiv_incidence_per_1000_uninfected_15_49 | 0.190398 | -0.00454 | 0.352067 |
| hiv_incidence_per_1000_uninfected_15_49 | 0.132002 | -0.08412 | 0.322205 |
| hiv_incidence_per_1000_uninfected_15_49 | -0.06117 | -0.19581 | 0.12315 |
| hiv_incidence_per_1000_uninfected_15_49 | 0.126499 | -0.04439 | 0.327669 |
| hiv_incidence_per_1000_uninfected_15_49 | -0.47458 | -0.70033 | -0.18325 |
| hiv_incidence_per_1000_uninfected_15_49 | -0.00675 | -0.15079 | 0.161587 |
| malaria_incidence_per_1000_at_risk | -0.21546 | -0.41911 | 0.072528 |
| malaria_incidence_per_1000_at_risk | 0.94686 | 0.89912 | 0.975845 |
| malaria_incidence_per_1000_at_risk | -0.32009 | -0.59661 | -0.08432 |
| malaria_incidence_per_1000_at_risk | -0.54518 | -0.68528 | -0.36934 |
| malaria_incidence_per_1000_at_risk | 0.660962 | 0.510427 | 0.778533 |
| malaria_incidence_per_1000_at_risk | -0.56702 | -0.69005 | -0.4143 |
| malaria_incidence_per_1000_at_risk | -0.60531 | -0.7156 | -0.46623 |
| malaria_incidence_per_1000_at_risk | -0.48336 | -0.58654 | -0.35982 |
| malaria_incidence_per_1000_at_risk | 0.551094 | 0.386134 | 0.689858 |
| malaria_incidence_per_1000_at_risk | 0.36671 | 0.150281 | 0.537873 |
| malaria_incidence_per_1000_at_risk | 0.470879 | 0.293277 | 0.619069 |
| hiv_prev_rate_female_per_100k | -0.19487 | -0.37465 | 0.062916 |
| hiv_prev_rate_female_per_100k | 0.184143 | -0.08968 | 0.463484 |
| hiv_prev_rate_female_per_100k | 0.180201 | -0.06172 | 0.409914 |
| hiv_prev_rate_female_per_100k | -0.31674 | -0.53694 | -0.0479 |
| hiv_prev_rate_female_per_100k | 0.336581 | 0.122516 | 0.511491 |
| hiv_prev_rate_female_per_100k | 0.275231 | -0.00324 | 0.512447 |
| hiv_prev_rate_female_per_100k | 0.036163 | -0.17794 | 0.322323 |
| hiv_prev_rate_female_per_100k | 0.143993 | -0.027 | 0.326515 |
| hiv_prev_rate_female_per_100k | -0.54322 | -0.75573 | -0.23705 |
| hiv_prev_rate_female_per_100k | -0.03098 | -0.19731 | 0.157049 |
| malaria_prev_rate_female_per_100k | -0.29299 | -0.54318 | -0.07153 |
| malaria_prev_rate_female_per_100k | -0.51178 | -0.65054 | -0.34784 |
| malaria_prev_rate_female_per_100k | 0.596466 | 0.450807 | 0.721503 |
| malaria_prev_rate_female_per_100k | -0.5529 | -0.67851 | -0.40237 |
| malaria_prev_rate_female_per_100k | -0.56669 | -0.67511 | -0.43739 |
| malaria_prev_rate_female_per_100k | -0.42589 | -0.52682 | -0.31331 |
| malaria_prev_rate_female_per_100k | 0.542098 | 0.402803 | 0.668273 |
| malaria_prev_rate_female_per_100k | 0.34781 | 0.149107 | 0.510305 |
| malaria_prev_rate_female_per_100k | 0.470278 | 0.311223 | 0.603765 |
| gdp_per_capita_ppp | 0.742117 | 0.632538 | 0.867264 |
| gdp_per_capita_ppp | -0.50494 | -0.74736 | -0.29789 |
| gdp_per_capita_ppp | 0.473272 | 0.24526 | 0.708126 |
| gdp_per_capita_ppp | 0.641268 | 0.442729 | 0.852144 |
| gdp_per_capita_ppp | 0.633472 | 0.47616 | 0.857534 |
| gdp_per_capita_ppp | -0.36663 | -0.58131 | -0.1884 |
| gdp_per_capita_ppp | -0.20826 | -0.51018 | -0.01743 |
| gdp_per_capita_ppp | -0.04589 | -0.50552 | 0.305679 |
| hdi | -0.79909 | -0.87478 | -0.69507 |
| hdi | 0.845058 | 0.772914 | 0.89767 |
| hdi | 0.903618 | 0.855597 | 0.938163 |
| hdi | 0.793553 | 0.732627 | 0.846992 |
| hdi | -0.40203 | -0.60307 | -0.15587 |
| hdi | -0.37919 | -0.62181 | -0.1819 |
| hdi | -0.12949 | -0.37404 | 0.154965 |
| tfr_children_per_woman | -0.807 | -0.85898 | -0.73883 |
| tfr_children_per_woman | -0.85266 | -0.89846 | -0.78999 |
| tfr_children_per_woman | -0.68828 | -0.76056 | -0.61213 |
| tfr_children_per_woman | 0.420769 | 0.16924 | 0.624067 |
| tfr_children_per_woman | 0.496025 | 0.303202 | 0.658398 |
| tfr_children_per_woman | 0.210435 | -0.03708 | 0.435589 |
| female_primary_completion_rate | 0.889776 | 0.856057 | 0.920278 |
| female_primary_completion_rate | 0.638637 | 0.569186 | 0.704764 |
| female_primary_completion_rate | -0.30891 | -0.49295 | -0.096 |
| female_primary_completion_rate | -0.45789 | -0.60001 | -0.32139 |
| female_primary_completion_rate | -0.13851 | -0.3346 | 0.081592 |
| female_secondary_gross_enrol | 0.819003 | 0.773617 | 0.864205 |
| female_secondary_gross_enrol | -0.41872 | -0.59549 | -0.20954 |
| female_secondary_gross_enrol | -0.40013 | -0.59665 | -0.23675 |
| female_secondary_gross_enrol | -0.22928 | -0.43033 | 0.012363 |
| female_tertiary_gross_enrol | -0.48241 | -0.64683 | -0.26955 |
| female_tertiary_gross_enrol | -0.32793 | -0.54769 | -0.16315 |
| female_tertiary_gross_enrol | -0.33342 | -0.49768 | -0.10366 |
| female_lfp | -0.01914 | -0.25153 | 0.222209 |
| female_lfp | 0.409128 | 0.201044 | 0.588776 |
| temp_mean_c | 0.034727 | -0.15594 | 0.21611 |

Supplementary Table S7B: Pearson coefficients were calculated from 1,092 observations representing 52 countries with 21 annual observations per country. Confidence limits are the 2.5th and 97.5th percentiles from 10,000 bootstrap replicates in which countries, rather than individual country-year rows, were sampled with replacement (random seed = 42). An interval excluding zero indicates that the bootstrap interval did not cross zero; no row-level p-values were calculated. Intervals are conditional on the completed imputed dataset and do not propagate imputation uncertainty.

END…
